# 

**Published:** 1917-11-17

**Authors:** 


### CONTENTS.

RATES OF SUBSCRIPTION: Twelve months, 6/6; Six months, 4/-; three months, 2/-, post free to any address in the United Kingdom. Abroad, Twelve months, 8/8; Six months, 5/-; Three months, 2/6, post free. THE HOSPITAL "Index" to Volume LXII., April 1917 to September 1917, is now ready, price 2½d. per copy, post free. Address The Manager, THE HOSPITAL, "The Hospital" Building, 28/29 Southampton Street, Strand, London, W.C. 2.

### Making Use of Smallpox Hospitals.

It has been suggested that the smallpox hospital recently provided by the Swansea Corporation should be used as an open-air residential school, a purpose which would enable the building to be released at very short notice if required for smallpox cases. The Inspector of the Board of Education has expressed the view that the Board of Education would agree to using the building for such a purpose, and in these circumstances the Swansea Corporation is asking the Local Government Board to sanction the proposal.

### HOSPITAL AND INSTITUTIONAL RESULTS.

**City of London Hospital for Diseases of the Chest, Victoria Park.**—The committee and the secretary, Mr. George Watts, are to be congratulated on the attractive appearance of the report; they evidently understand the value of a nice-looking report in propaganda. The contents give evidence of successful and economical work. The list of named beds and cots is very modest, and the endowment fund correspondingly low.

**The Infants' Hospital.**—£2,100 is rather a heavy loan to carry for a hospital with an income of less than £3,000 a year. This appears to be the result of accumulated deficits on current working. The committee might consider the advisability of taking small fees from the parents or friends of patients towards the cost of maintenance or medicines.

**Royal Victoria Infirmary, Newcastle-on-Tyne.**—The striking feature about this report is as always the splendid sum recorded as the contribution of the workmen of the neighbourhood to their hospital. Last year the total was £29,140, as against £24,722 in 1915, and this out of a total income of £93,418. The generosity of the workpeople was not in the least degree affected by the National Health Insurance Act. The result is a tribute also to present and past organisation.

**Essex County Hospital, Colchester.** — The report is confined to bare essentials, and even the cover is used for the publication of part of the accounts. No list of subscribers is issued this year. Letters of recommendation will not be sent to subscribers unless specially asked for. There was a deficit of £242 on the year's working.

**South Devon and East Cornwall Hospital, Plymouth.** — The report is abridged, but still runs to ninety-five pages. The contents table refers to the pages that are there as well as the pages that are not there. The blanks are rather reminiscent of the mysterious gaps in "Tristram Shandy," but any curious member of the public may be enlightened on application to the secretary. There was a difference on the wrong side between ordinary income and ordinary expenditure, but the legacies more than made this good.

**Miller Hospital, Greenwich Road, S.E.** —The taking over of Lewisham Infirmary by the military authorities has increased the call of the neighbourhood on the resources of the hospital; ten emergency beds and cots were added, and there was an occupancy of seventy-one in seventy-six available. This quick filling of beds is a sign of good management. Although legacies amounted to £100 only, there was a balance to the good on the income and expenditure account.

**Liverpool Royal Infirmary.**—This is one of the few provincial hospitals making a statistical return in the form required of Metropolitan hospitals by the Central Funds. Why not go a step further and present an income and expenditure account and a balance-sheet according to the same model? The present form would require but little modification. Amongst the receipts are contributions in respect of military and civil patients amounting to £7,245.

**Asylums.**—Reports have been received from the following: County of Salop Asylum, Carmarthen Asylum, Borough of Leicester Mental Hospital, Down District Asylum, Hereford County Asylum, and West Sussex County Asylum. The weekly cost of maintenance per patient varies from 9s. 10½d. at Salop to 13s. 10d. at Carmarthen. We notice that the weekly cost at the County of Salop Asylum was lower by 1s. 1d. than it was in 1913, the year before the war.

### Matrons' and Sisters' Appointments.

BRIGHTON POOR-LAW INFIRMARY.—Miss Maude Hexamer has been appointed night sister. She was trained at the Central London Infirmary, Hendon, and has been ward sister at Bethnal Green Infirmary, Shirley Warren Union Infirmary, and Bristol Fever Hospital.

GRESSENHALL INFIRMARY, DEREHAM.—Miss Laura S. Withers has been appointed head nurse. She was trained at the Union Infirmary, Norwich, and holds the C.M.B. certificate.

HACKNEY UNION INFIRMARY, HOMERTON.—Miss Mary Baird has been appointed superintendent nurse. She was trained at Salford Union Infirmary, and has since held various appointments. Miss Rose Bright has been appointed charge sister at this institution. She was trained at Greenwich Union Infirmary. Miss Annie E. Minns has been appointed ward sister. She was trained at Great Yarmouth Union Infirmary.

ISOLATION HOSPITAL, BEDWELLTY, MON.—Miss Lillian Evans has been appointed sister in charge. She was trained at the Borough Isolation Hospital, Eastbourne, and King Edward VII.'s Hospital, Cardiff.

KILSYTH FEVER HOSPITAL.—Nurse Jessie B. Henderson has been appointed matron. She was trained at Belvidere Fever Hospital, Glasgow, and has since been charge nurse at Arbroath Public Health Hospital.

VICTORIA HOSPITAL, SWINDON.—Miss Dorothy Horton has been appointed sister. She was trained at the Bath Royal United Hospital, where she has since been staff nurse and acting sister.

SELLY OAK INFIRMARY, BIRMINGHAM.—Miss Margaret Lawrence and Miss J. B. Traish have been appointed ward sisters. They were both trained at Southwark Infirmary.

UPPER EDMONTON, GENERAL MILITARY HOSPITAL.—Miss Ethel May Willcock has been appointed sister. She was trained at the Guest Hospital, Dudley, and has since held various appointments at the Eastern Hospital, Homerton, at Monsall Fever Hospital, Manchester, and at the Louise Margaret Hospital, Aldershot.

WELL LANE MILITARY HOSPITAL, WEST DIDSBURY.—Miss Esther Wolstenholme has been appointed sister. She was trained at Monsall Fever Hospital and Salford Royal Hospital, was attached to the private staff of Preston Royal Infirmary, and has since been sister at Monsall Hospital, night sister at Queen Mary's Hospital for the East End, London, and day and night sister at Longford Hall Auxiliary Military Hospital, Stretford, Manchester.

WILLESDEN INFIRMARY, ACTON LANE.—Miss Mary Susan Blandford, Miss Ethel Maude Mackey, and Miss Elsie Chequer have been appointed ward sisters. They were all trained at Willesden.

SHEFFIELD CHILDREN'S HOSPITAL.—Miss Harriet McNalley has been appointed sister. She was trained at Bury Infirmary, and has been staff nurse at the Oxford Eye Hospital, sister of the military wards and out-patient department at the Royal Eye Hospital, Manchester, and sister at the Lord Mayor Treloar Cripples' Hospital, Alton. She has also done private nursing.

ISOLATION HOSPITAL, RAWMARSH.—Mrs. M. Haywood has been appointed matron. She was trained at the Royal Victoria Infirmary, Newcastle-on-Tyne, and has been charge nurse at the Isolation Hospital, Wallsend, sister and deputy matron at the Harrogate and Knaresborough Isolation Hospital, and matron at the Isolation Hospital, Llantrisant, South Wales.

BIRMINGHAM MUNICIPAL CRECHE.—Miss Beatrice Gebhard has been appointed matron. She was trained at Firvale Hospital, Sheffield, and has held the posts of staff nurse at Whipps Cross Infirmary, Leytonstone, ward sister at Ecclesall Infirmary, Sheffield, night superintendent at the Anlaby Road Infirmary, Hull, and home sister and assistant matron at Township Infirmary, Leeds.

ST. MARK'S HOSPITAL, E.C.—Miss E. M. Streeter has been appointed night sister. She was trained at the Royal West Sussex Hospital, Chichester, where she later temporarily filled sister's duties.

AUXILIARY MILITARY HOSPITAL, LEATHERHEAD.—Miss E. C. Dawson has been appointed night sister. She was trained at the Walsall and District General Hospital, Staffs, and has since nursed at the Johnson Hospital, Spalding, and at Townley's Military Hospital, Farnworth, near Bolton.

AUXILIARY HOSPITAL, BLACKLEY, MANCHESTER.—Miss Marion Smailes has been appointed matron. She was trained at the Derbyshire Royal Infirmary, and has been matron at the Salford Day Nursery and sister at Sedgley Hall Red Cross Hospital, Manchester.

WESTERN FEVER HOSPITAL, SEAGRAVE ROAD, FULHAM.—Miss Alice M. Mayhew has been appointed sister. She was trained at Kensington Infirmary, London.

JESSOP HOSPITAL FOR WOMEN, SHEFFIELD.—Miss Amy Clapham has been appointed sister. She was trained at the General Infirmary, Leeds, later taking sister's duties there.

ST. BARTHOLOMEW'S HOSPITAL, ROCHESTER.—Miss Beatrix Hirsch has been appointed night sister. She was trained at St. Bartholomew's Hospital, London, has been sister at the Military Orthopædic Hospital, Hammersmith, and has worked in Roumania.

MILL BAY AUXILIARY HOSPITAL, PLYMOUTH.—Mrs. D. Unwin has been appointed sister-in-charge. She was trained at the Royal Southern Hospital, Liverpool. She has since worked in the theatre at King George's Hospital, Waterloo, has been dispenser and theatre sister at Victoria Military Hospital, Keighley, ward sister at No. 5 War Hospital, Exeter, and sister at Mill Bay Hospital.

ROYAL SURREY COUNTY HOSPITAL, GUILDFORD. — Miss Dorothy Newberry has been appointed home sister. She was trained at the London Hospital, later being ward sister there.

ST. LEONARD'S HOSPITAL, SUDBURY, SUFFOLK.—Miss H. G. Newman has been appointed sister. She was trained at Chelsea Infirmary.

ROYAL LIVERPOOL COUNTRY HOSPITAL FOR CHILDREN, HESWALL.—Miss Lily Barraclough has been appointed ward sister. She was trained at Chester Royal Infirmary and at the Children's Hospital, Gateshead, and has worked at the 1st Northern General Hospital, Newcastle-on-Tyne.

**NOTE.—Particulars of Appointments for insertion in this column will be welcomed.**

### Meetings and Functions.

THE governors of St. George's Hospital, at the next general court of governors, will be asked to appoint Lieut.-Colonel R. M. Carter, I.M.S., of Mesopotamia fame, an honorary life-governor.

THE Harben Lectures at the Royal Institute of Public Health, announced to be held on November 26, 27, and 28, have been postponed owing to Dr. Alexis Carrel having been kept by official demands in America.

THE Ministry of Food have arranged a lecture at Grosvenor House on November 19, at 11.30 A.M., when Dr. Campbell will deliver short addresses on the potato position and the use of potatoes as helping in the conservation of cereal crops.

THE Lady Priestley Memorial Lecture under the auspices of the National Health Society will be given this year on Wednesday, November 21, at 3 P.M., in the Robert Barnes Hall, 1 Wimpole Street, W. Sir Arthur Newsholme, K.C.B., M.D., F.R.C.P., Medical Officer of the Local Government Board, is the lecturer, his subject being "The Child and the Home." The chair will be taken by the Right Hon. Hayes Fisher, M.P., President of the Local Government Board, and the meeting will be addressed by, amongst others, Sir James Crichton Browne, M.D., F.R.S.

